# Probable Primary Cutaneous CD8+ Aggressive Epidermotropic Cytotoxic T-cell Lymphoma: A Case Report of a Diagnostic Challenge

**DOI:** 10.7759/cureus.44375

**Published:** 2023-08-30

**Authors:** Marcela Velarde Loya, Monica G Millan Reza, Mariana Olaya Cordova, Zaira D Chavéz López

**Affiliations:** 1 Internal Medicine Residency, Hospital General Presidente Lázaro Cárdenas, Instituto de Seguridad y Servicios Sociales de los Trabajadores del Estado (ISSSTE), Chihuahua, MEX; 2 Dermatology, Universidad Nacional Autónoma de México (UNAM), Chihuahua, MEX

**Keywords:** epidermotropism, ki67, cytotoxic lymphocytes, cd8, cutaneous lymphoma

## Abstract

Primary cutaneous CD8+ aggressive epidermotropic cytotoxic T-cell lymphoma is a rare variety of cutaneous lymphoma. This subtype has an aggressive and quickly progressive clinical course with a survival time of 32 months from the commencement of skin lesions. This article describes a probable case of primary cutaneous CD8+ aggressive epidermotropic cytotoxic T-cell lymphoma in a 63-year-old female, which manifested as diffuse non-pruritic erythematous plaques and nodules. The diagnosis of this possible entity was aided by the histopathological and immunohistochemical findings, while immunohistochemistry for T-cell receptor (TCR) gamma/delta could not be done.

## Introduction

Primary cutaneous lymphomas (PCLs) are non-Hodgkin's lymphomas that develop in the skin with no signs of any extracutaneous disease at the time of diagnosis [1,2]. They are a diverse set of lymphoproliferative processes that include subtypes B and T, in which neoplastic lymphocytes have a specific affinity for skin tissue [2].

Mycosis fungoides is the most frequent subtype of cutaneous lymphoma, accounting for about 75% of all PCLs [1]. The majority have a T-helper memory lymphocyte phenotype (CD3+, CD4+, and CD45R0+) [2], while only a few have a CD8+ phenotype, with some having an indolent clinical course and others an aggressive one [1,3].

In the international medical literature, aggressive cutaneous epidermotropic cytotoxic T-cell lymphoma accounts for less than 1% of all T-cell lymphomas. It has been recorded more frequently in men, with a mean starting age of 77 years [3]. They are a diverse set of lymphoproliferative processes that arise from mature T cells producing one or more of the cytotoxic granules TIA-1 (T-cell intracellular antigen-1), granzyme B, and perforin [2,4]. Because of its rarity, little is known about its pathophysiology as well as the cellular and genetic factors involved [3].

## Case presentation

**Figure 1:**
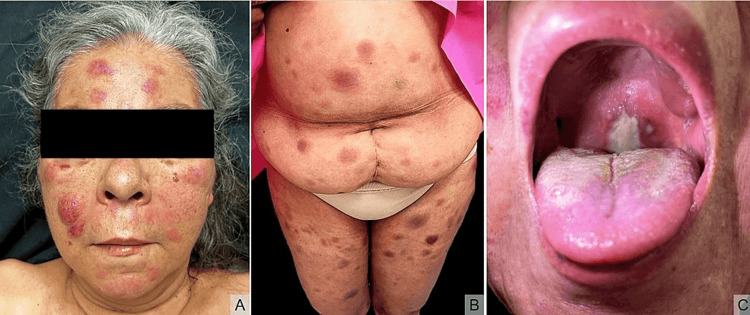
Multiple erythematous nodules on the face (A) and abdomen and lower extremities (B). A soft palate ulcer (C).

A 63-year-old female patient presented with multiple well-defined erythematous nodules, some of them with central ulceration, ranging in size from 1 to 3 cm, present over the face, abdomen, and four extremities (Figure 1). The lesion had started as erythematous macules four months prior to consultation. They were neither pruritic nor linked with systemic symptoms. Blood studies showed elevation of alkaline phosphatase, lactic dehydrogenase, and tumor markers CA 15-3, CA 19-9, and CA 125.

**Figure 2:**
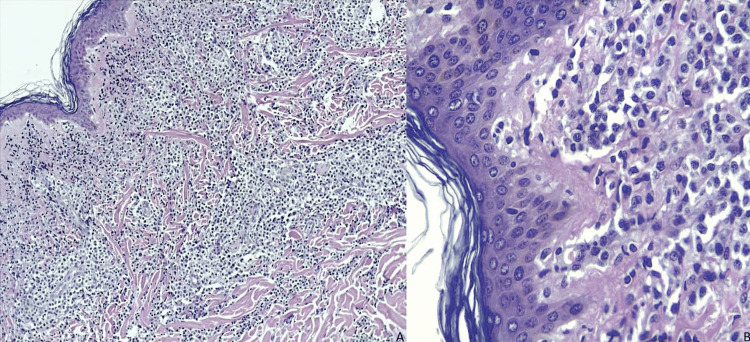
Focal epidermotropism and dense mononuclear-type proliferation grouped in a band at the papillary and reticular dermis (A). Atypical lymphocyte infiltrate with hyperchromatic nuclei (B).

**Figure 3:**
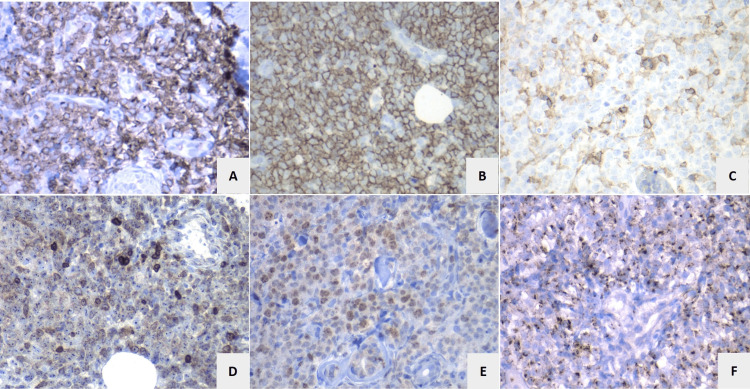
CD3 (A), CD8 (B), CD4 (C), BCL-2 (D), Ki-67 (E), and granzyme (F) were all positive in the tumor cell population. CD: cluster of differentiation; BCL-2: B-cell lymphoma 2.

An incisional biopsy of the lesion in the leg was performed due to the suspicion of cutaneous lymphoma. Biopsy revealed a focal epidermotropic and band-like dermal lymphocytic infiltrate, composed of medium-sized atypical lymphocytes, extending to subcutaneous tissue. There was no evidence of angioinvasion or adnexal damage (Figure 2). Immunohistochemistry demonstrated a positive CD3, CD4, CD8, TIA1, and granzyme B (cytotoxic granules) immunophenotype (Figure 3), with loss of CD20, CD30, BCL-6, and CD45RO. Approximately 60% of cells were Ki67+. Immunohistochemistry for T-cell receptor (TCR) gamma/delta, BethaF-1, and CD45RA was not available. Histological findings, in conjunction with the immunohistochemistry profile, were consistent with a rare subtype of peripheral T-cell PCL.

The patient had extracutaneous progression shortly after the diagnosis, including involvement of the oral mucosa and gradually progressive dysphagia, for which she was admitted to the hospital by the hematology service to evaluate the initiation of a chemotherapy regimen with CHOP (combination of cyclophosphamide, adriamycin, vincristine, and prednisone). Nevertheless, the patient passed away shortly after being admitted as a result of acute respiratory failure syndrome.

## Discussion

Primary cutaneous CD8+ aggressive epidermotropic cytotoxic T-cell lymphoma (Berti's lymphoma) was initially identified in 1999 by Berti et al. [2]. This form of lymphoma accounts for 1% of all cutaneous T-cell lymphoma cases, with slightly more than 30 cases recorded in the literature [5,6]. 

It appears as localized or diffuse eruptive papules, superficial hyperkeratotic annular plaques or nodules, and ulcerating tumors on the skin. They can spread to other organs such as the lung, testicles, central nervous system, and oral mucosa without involving the lymph nodes [2,3,7].

The most common cutaneous lymphomas are low-grade, with an indolent clinical course [1]. There are some unusual entities with aggressive behavior, such as gamma-delta lymphomas, natural killer (NK)/T-cell lymphoma, aggressive CD8+ epidermotropic T lymphoma, or primary cutaneous large B-cell lymphoma, leg type [3,5].

Presentation and clinical behavior distinguish this cutaneous lymphoma from other cutaneous lymphomas. CD8+ mycosis fungoides [8,9], gamma-delta mycosis fungoides, lymphomatoid papulosis subtype D, and primary cutaneous T-cell lymphoma, not otherwise specified (NOS) should all be considered [5].

In nearly every case reported in medical literature, histopathology revealed an epidermotropic infiltrate with a pagetoid or band pattern of atypical monomorphic T-cells of varying size, with the following findings in immunohistochemistry: CD3+, CD4-, CD8+, CD7-/+, CD45RA+, with cytotoxic granules (TIA-1, granzyme B, perforin) [2,7,10]. It is usual for cutaneous adnexal structures to be invaded and destroyed [4,9]. 

Cases with positivity for different antibodies have been reported in the literature. The most frequent immunoprofile has positive results for CD3, TIA-1, and granzyme, in addition to the presence of CD8+ lymphocytes, and negative results for CD4 and CD5. In the case of our patient, the findings were consistent with a rare subtype of peripheral T-cell PCL, of which Berti's lymphoma was our primary differential diagnosis due to the clinical presentation and histopathological and immunohistochemical findings. However, these same findings can overlap with the other rare subtypes in the WHO classification, like gamma/delta lymphomas, as well as primary cutaneous T-cell lymphomas NOS [3,11].

There are no pathognomonic criteria for this entity at this time, requiring the performance of genetic and immunohistochemical tests, which are limited in various regions of the world, including Mexico. This case exemplifies the clinical challenge of diagnosing and classifying these types of cutaneous lymphomas [3,11,12].

As of today, no therapeutic guidelines or controlled trials that evaluate the efficacy of traditional treatments for these subtypes of cutaneous lymphoma have been published. The prognosis is very poor, with a median survival of only 32 months from the onset of skin lesions [5,7,9,13]. The worst prognosis is observed with a Ki-67 index greater than 60% [3]. Since it is not a well-known entity, dismal results, recurrence, and a high prevalence of adverse effects may be shown [3,6].

## Conclusions

Primary cutaneous CD8+ aggressive epidermotropic cytotoxic T-cell lymphoma sets a little-known scenario, as it displays a quick and aggressive behavior. Its diagnosis can be difficult; thus, it is critical to include this condition as a differential diagnosis in the early workup by a multidisciplinary team of dermatologists, dermatopathologists, hematologists, radiation oncologists, and hematopathologists.
